# Protective effects of isorhamnetin against H_2_O_2_-induced oxidative damage in HaCaT cells and comprehensive analysis of key genes

**DOI:** 10.1038/s41598-023-27575-7

**Published:** 2023-02-13

**Authors:** Wen Hu, Jingzhan Zhang, Hongjuan Wang, Mengmeng Guan, Leheng Dai, Jun Li, Xiaojing Kang

**Affiliations:** 1 Department of Dermatology and Venereology, People’s Hospital of Xinjiang Uygur Autonomous Region, Urumqi, Xinjiang China; 2 Xinjiang Clinical Research Center for Dermatologic Diseases, Urumqi, Xinjiang China; 3 Xinjiang Key Laboratory of Dermatology Research (XJYS1707), Urumqi, 830000 Xinjiang China

**Keywords:** Experimental models of disease, Outcomes research

## Abstract

Isorhamnetin (ISO) is a methylated flavonol present in the leaves, flowers, and fruits of many plants with antitumour, anti-inflammatory, antioxidant, and anti-apoptotic properties. ISO has been suggested as the active substance in *Vernonia anthelmintica* (L.) to treat vitiligo. However, the mechanisms underlying its effects remain unclear. In this study, human keratinocytes (HaCaT cells) were pre-treated with or without ISO and then stimulated with hydrogen peroxide (H_2_O_2_) to generate oxidative damage. Pre-treatment with ISO increased HaCaT cell viability, reduced malondialdehyde content, and enhanced superoxide dismutase activity, resulting in a reduction in the loss of mitochondrial membrane potential, improved cell morphological damage, and apoptosis inhibition. Furthermore, we identified 51 significantly dysregulated differentially expressed genes (DEGs) of HaCaT cells treated with ISO using RNA-sequencing. Enrichment analysis using Gene Ontology and Kyoto Encyclopedia of Genes and Genomes databases indicated that the protective effect of ISO could be related to its effects on the Wnt signalling pathway. Our study provides novel insights into key gene regulation in the progression of oxidative damage and the mechanisms of action of ISO.

## Introduction

Vitiligo is a common depigmentation disorder, clinically characterised by melanocyte destruction and melanosome loss, with a prevalence of 0.5–2% worldwide^1^. Vitiligo is often accompanied by various autoimmune diseases such as hyperthyroidism, diabetes, alopecia areata, psoriasis, and asthma^2,3^. It can occur in any part of the body, especially the exposed and wrinkled parts that are prone to friction and exposed to sunlight, and the palm plantar, mucosa, and retina can also be involved. Vitiligo affects a person’s appearance and can seriously impact their work and personal relationships; patients with vitiligo often suffer from social discrimination, causing anxiety or depression and other mental disorders, as the disfiguring spots hamper their social interactions^2^. Moreover, the disease is persistent and readily relapses; further, the treatment of this condition is difficult^4^. Although the exact cause of, or mechanism underlying, vitiligo remains mostly unknown, many theories have been proposed to explain the mechanism of pigmentation loss, such as autoimmunity, neural and genetic disease, impaired melanocyte migration and/or proliferation, and oxidative stress^5^. Oxidative stress is considered one of the most crucial initiators of vitiligo; it is involved in the development of vitiligo and is one of the factors responsible for the induction of the autoimmune response associated with the condition^6,7^.

Oxidative stress disturbs redox homeostasis and causes an imbalance between pro-oxidants and antioxidants^8^. Oxidative stress in tissues and cells results from excessive levels of reactive oxygen species (ROS). Oxidative stress-induced damage causes excessive aggregation of hydrogen peroxide (H_2_O_2_) in the epidermis of vitiligo patients, and a large increase in intracellular ROS levels. Oxidative stress can further cause oxidative damage to nucleic acids, proteins, and lipids, subsequently resulting in local melanocyte damage, apoptosis, and induction of an autoimmune response to attack more melanocytes, which causes progressive skin pigment loss^9^. Accumulating evidence indicates that keratinocytes are vital for the progression of vitiligo^10^. Although melanocytes are the main target cells of vitiligo, keratinocytes are also affected by the melanocyte destruction, which interrupts a vital nexus in the mechanisms underlying vitiligo development and progression^11^. Keratinocytes are mediators of detrimental oxidative stress, numerically dominate the cell composition of the epidermis, and directly interact with melanocytes. They can also transfer H_2_O_2_ to neighbouring melanocytes, which aggravates ROS accumulation and induces melanocyte destruction, thereby promoting vitiligo^12^. Oxidative stress has also been shown to induce damage to keratinocytes. Melanocytes and keratinocytes constitute the epidermal melanin units, which are closely related in structure and function, and play an important role in the transmission of melanosomes^13^. Keratinocytes, as mediators of oxidative stress, secrete cytokines to recruit autoreactive T cells that interfere with signal transduction in melanocytes, subsequently inducing melanocyte destruction^10–12^.

Isorhamnetin (ISO, 3-methyl quercetin; molecular formula: C_16_H_12_O_7_) is a methylated flavonol present in the leaves, flowers, and fruits of many plants^14,15^. ISO has numerous biological activities, including antitumour, anti-inflammatory, antioxidant, and anti-apoptotic properties^16–18^. Increasing evidence indicates that ISO from the plant material of *Schisandra rubriflora*, and its phenolic and anthocyanin compounds, has high antioxidant potential^19,20^. Moreover, the presence of phenolic compounds in bee bread significantly reduced the levels of oxidised-low-density lipoprotein (oxLDL) and malondialdehyde (MDA). It also significantly increased aortic antioxidant activities such as those of superoxide dismutase (SOD) and glutathione peroxidase (GPx)^21^. In addition, recent findings indicate that ISO is the main active substance in *Vernonia anthelmintica* (L.), and significantly increases the expression of melanin-biosynthetic genes (*MC1R*, *MITF*, *TYR*, *TYRP1*, and *DCT*) and tyrosinase activity in B16F10 cells^22^. *Vernonia anthelmintica* (L.), a traditional herbal medicine that is only grown in Kashgar, Aksu, and other areas in Xinjiang and India, is the most common herbal medicine for the treatment of vitiligo^23^. However, its mechanism of action remains unclear owing to the complexity of multiple ingredients and the lack of research on its chemical composition. In our previous study^24^, we identified 38 flavonoids from the seeds of *V. anthelmintica* (L.) using selective response/multiple response monitoring technology, and the content of total flavonoids was 0.97 g/100 g, among which ISO was present at a concentration of 93.23 ± 9.55 ng/g.

Currently, the pathogenesis of vitiligo is not well understood, treatment is difficult, and relapse is common^25^. Several treatment options aim to restore pigmentation, including excimer lasers, vitamin D analogues, and steroid therapy^26^. Unfortunately, these treatments are not widely employed because they induce long-term side effects^27^. Thus, several studies have focused on the identification of novel therapeutic medicines and on the assessment of multiple targets to explain the complex network of vitiligo-associated mechanisms. The aim of this study was to investigate the antioxidant activity and identify key genes to help reduce H_2_O_2_-induced oxidative damage in HaCaT cells (a human keratinocyte cell line) after ISO treatment. The results of the current study are expected to contribute to the development of ISO as a source of antioxidants and provide a theoretical basis for the targeted therapy of vitiligo.

## Results

### ISO protects HaCaT cells from H_2_O_2_-triggered injury

**Figure 1:**
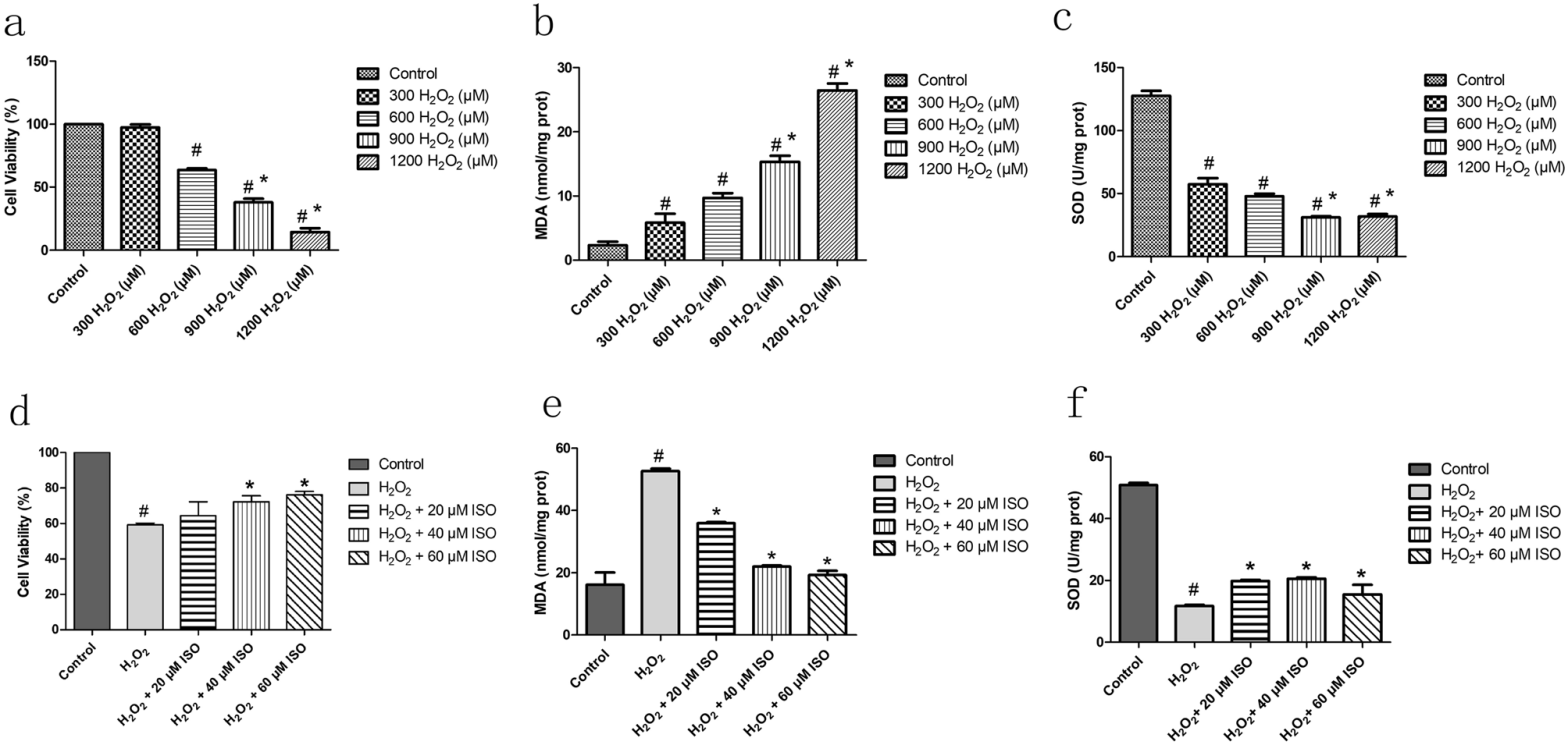
Effect of ISO on the cell viability, MDA content, and SOD enzyme activity after H_2_O_2_-treatment. The effect of H_2_O_2_ on (**a**) the cell viability and (**b**) MDA content and (**c**) SOD enzyme activity in HaCaT cells. Control, untreated HaCaT cells; The experimental groups, cells were treated with a concentration 300, 600, 900, 1200 μmol/L of the experimental sample for 24 h. The effect of ISO on (**d**) the cell viability, (**e**) MDA content, and (**f**) SOD enzyme activity in HaCaT cells. Control, untreated HaCaT cells; H_2_O_2_-alone group, HaCaT cells treated with 600 μM H_2_O_2_; all H_2_O_2_ + cells were treated with ISO 600 μM H_2_O_2_. Graphs show the mean ± SD values of three replication. One-way analysis of variance (ANOVA) followed by Tukey’s post-hoc test. # indicates significant differences (*p* < 0.05) from the control group. * indicates significant differences (*p* < 0.05) from the treatment of HaCaT cells with 600 μM H_2_O_2_ group.

The effects of different concentrations of H_2_O_2_ on the viability, MDA content, and SOD enzyme activity in HaCaT cells are shown in Fig. 1. The MDA content (Fig. 1b) was increased in a dose-dependent manner and the SOD level (Fig. 1c) was reduced significantly as the concentration of H_2_O_2_ increased. Cell viability (Fig. 1a) in the H_2_O_2_-alone group (300, 600, 900, or 1200 μM H_2_O_2_) was significantly reduced compared with that of the control group (no H_2_O_2_), indicating that H_2_O_2_ induced cytotoxic damage to HaCaT cells (*p* < 0.05). The half-maximal inhibitory concentration (IC_50_) value of H_2_O_2_ was approximately 600 μM. Based on the above results, 600 μM H_2_O_2_ treatment was selected to establish the oxidative-stress model of HaCaT cells.

The protective effect of ISO on the viability of HaCaT cells treated with H_2_O_2_ was detected using the CCK-8 assay. As shown in Fig. 1, as the concentration of ISO increased, the cell viability (Fig. 1d) in the experimental group (20, 40, or 60 μM ISO) became significantly higher than that in the H_2_O_2_-alone group. The expression levels of MDA (Fig. 1e) were higher in the H_2_O_2_-alone group than in the control group. Compared with the H_2_O_2_-alone group, the level of MDA in the samples pre-treated with ISO was significantly lower. The expression level of SOD (Fig. 1f) in the H_2_O_2_-alone group was significantly reduced compared with that in the control group, whereas the SOD level in cells pre-treated with ISO was significantly increased compared with that of the H_2_O_2_-alone group. These results indicate that ISO pre-treatment can significantly reduce the inhibitory effect of H_2_O_2_ on antioxidant enzymes.

### ISO protects HaCaT cells from H_2_O_2_-induced apoptosis

**Figure 2:**
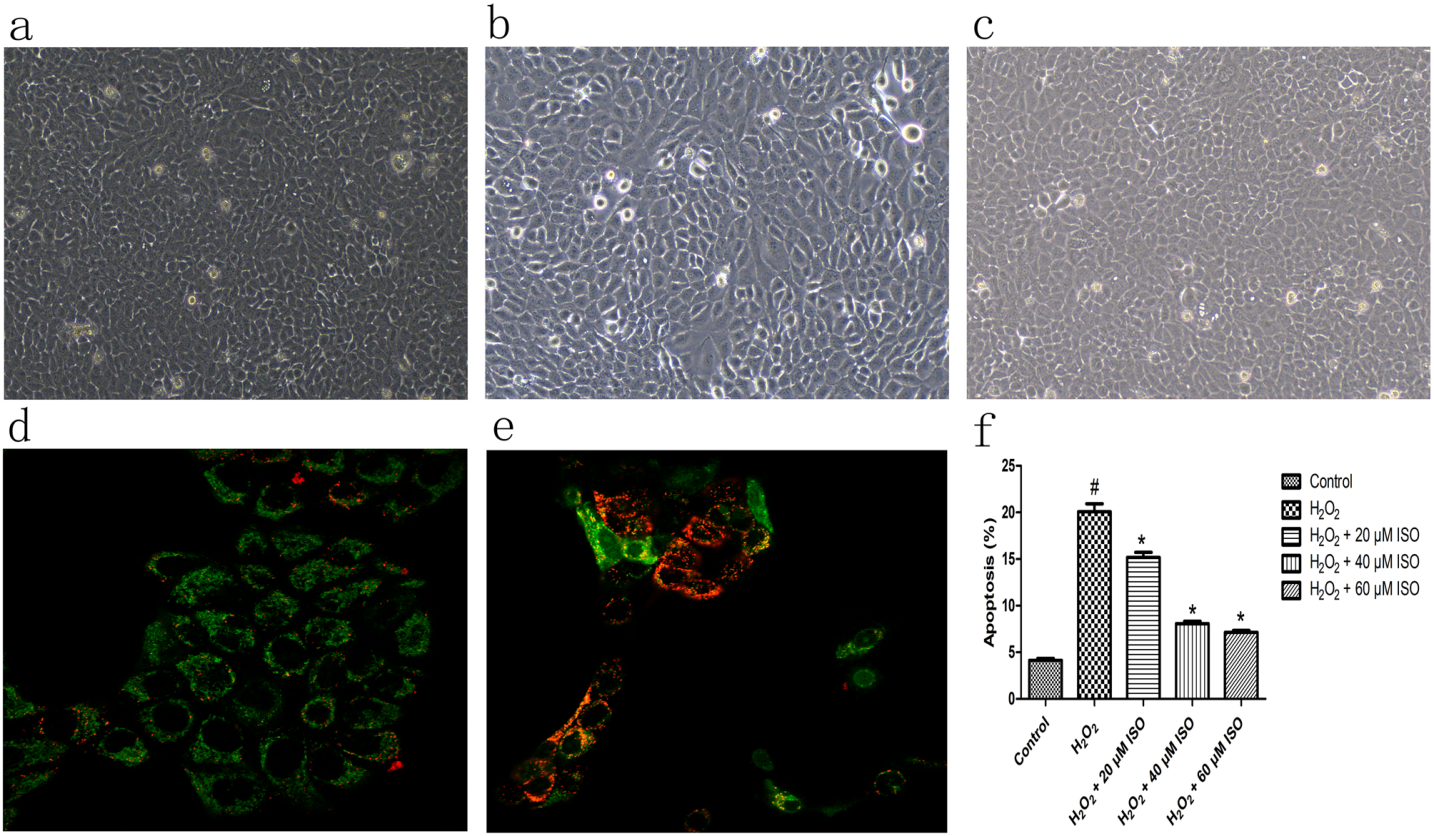
The effect of isorhamnetin (ISO) on the morphology of HaCaT cells injured by H_2_O_2_ (40 × magnification). (**a**) Untreated HaCaT cells showing a normal cell morphology. (**b**) HaCaT cells stimulated with 600 μM H_2_O_2_ showed increased apoptosis. (**c**) HaCaT cells treated with 600 μM H_2_O_2_ + 40 μM ISO group, compared with H_2_O_2_-only treatment, showing improvement in cell morphology and a reduction in the number of apoptotic cells. (**d**) Confocal microscopy images of HaCaT cells were treated with 600 μM H_2_O_2_ to detect mitochondrial permeability; weak red fluorescence and strong green fluorescence indicates disruption of mitochondrial membrane potential. (**e**) Confocal microscopy of cells treated with 600 μM H_2_O_2_ + 40 μM ISO treatment, showing stronger red fluorescence. (**f**) Apoptosis rate of cells treated with hydrogen peroxide as assessed by PI staining and flow cytometry. Control, untreated HaCaT cells; H_2_O_2_-alone group, HaCaT cells treated with 600 μM H_2_O_2_; all H_2_O_2_ + ISO were 600 μM H_2_O_2_. Graphs show the mean ± SD values of three replication. One-way ANOVA followed by Tukey’s post-hoc test. # indicates significant differences (*p* < 0.05) from the control group. * indicates significant differences (*p* < 0.05) from the treatment of HaCaT cells with 600 μM H_2_O_2_ group.

The effect of ISO on the morphology of HaCaT cells injured by H_2_O_2_ is shown in Fig. 2. Compared with the control group (Fig. 2a), treatment of HaCaT cells with 600 μM H_2_O_2_ (Fig. 2b) induced apoptosis; compared with the H_2_O_2_-alone group, cells pre-treated with 40 μM ISO (Fig. 2c) revealed improved cell morphology and a reduction in the number of apoptotic cells. Apoptosis is mediated via mitochondrial membrane permeabilisation events^18^. To investigate whether ISO can inhibit H_2_O_2_-induced apoptosis stemming from mitochondrial dysfunction, the effect of ISO on the mitochondrial membrane potential of HaCaT cells after H_2_O_2_-triggered injury was assessed. Confocal microscopy showed that the mitochondria of H_2_O_2_-induced cells emitted weak red fluorescence and strong green fluorescence (Fig. 2d), indicating that the mitochondrial membrane potential of cells was disrupted. However, after ISO pre-treatment, the red fluorescence was significantly higher than that in the H_2_O_2_-alone group (Fig. 2e), indicating that the increase in green fluorescence intensity was almost completely prevented by ISO. These results showed that ISO could reduce the loss of mitochondrial membrane potential in HaCaT cells with H_2_O_2_-triggered injury.

To investigate the effects of ISO on the H_2_O_2_-induced apoptosis of HaCaT cells, propidium iodide (PI) staining was performed, as shown in Fig. 2f. The apoptosis rates of the control group, H_2_O_2_-alone group, H_2_O_2_ + 20 μM ISO, H_2_O_2_ + 40 μM ISO and H_2_O_2_ + 60 μM ISO were 4.13 ± 0.21%, 20.07 ± 0.85%, 15.17 ± 0.55%, 8.07 ± 0.15% and 7.13 ± 0.21%, respectively. Thus, compared with the H_2_O_2_-alone group, cell apoptosis was alleviated in the groups pre-treated with ISO, in a dose-dependent manner.

### Differentially expressed genes (DEGs) and functional enrichment analysis

**Figure 3:**
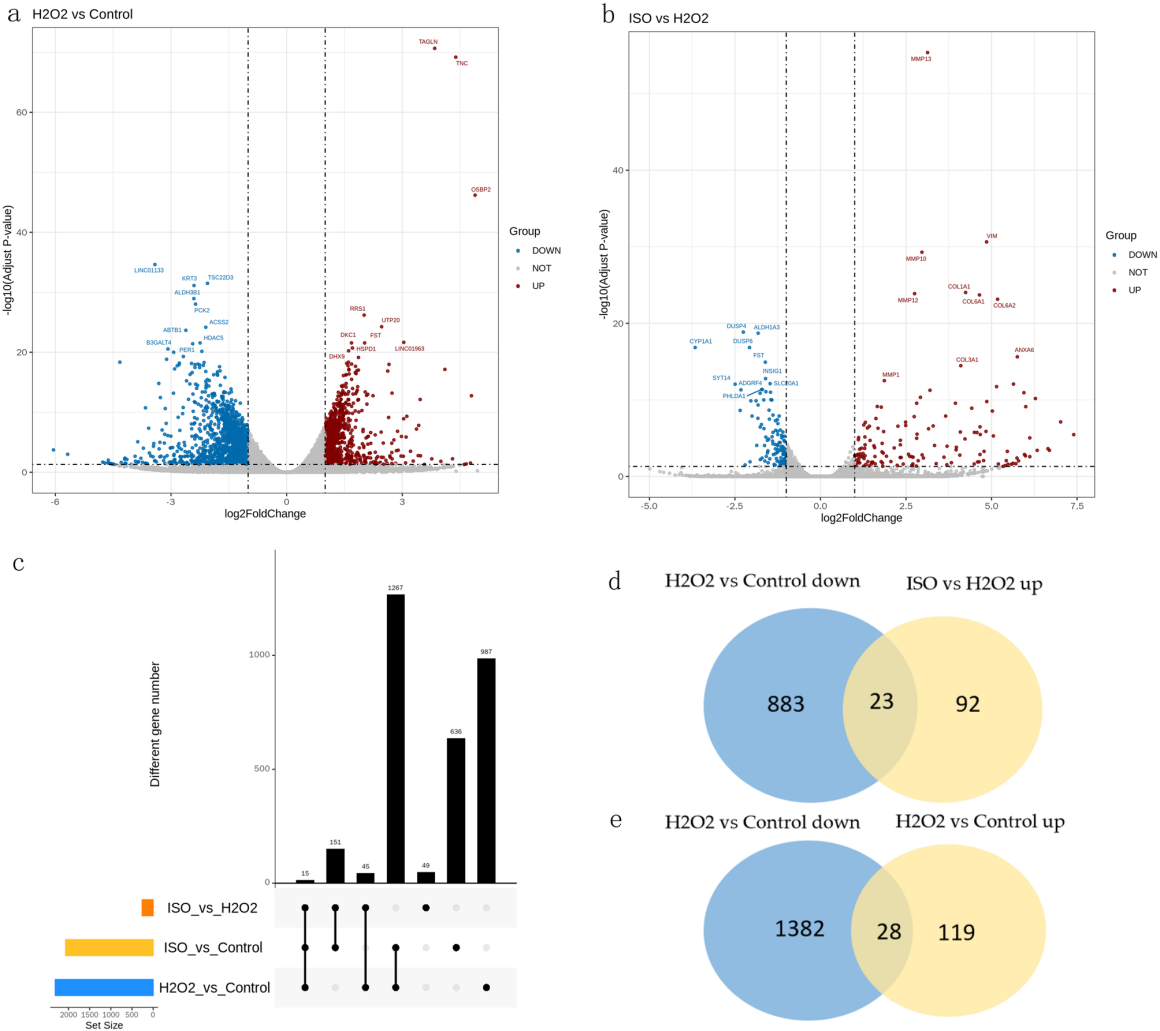
Differentially expressed genes (DEGs). (**a**,**b**) Volcano plot of gene expression profiles in the (**a**) control vs. H_2_O_2_ and (**b**) ISO vs. H_2_O_2_ comparisons. (**c**–**e**) Venn diagrams showing (**c**) unique or shared DEGs for those (**d**) upregulated in H_2_O_2_ vs. control and downregulated in H_2_O_2_ + ISO and genes that are (**e**) downregulated in H_2_O_2_ vs. control and upregulated in H_2_O_2_ + ISO. *H*_*2*_*O*_*2*_ hydrogen peroxide, *ISO* isorhamnetin, *control group* untreated HaCaT cells, *H*_*2*_*O*_*2*_* group* HaCaT cells treated with 600 μM H_2_O_2_ for 12 h, *ISO group* 600 μM H_2_O_2_ + 40 μM ISO.

We selected three groups of HaCaT cells (control, H_2_O_2_-alone, and H_2_O_2_ + 40 μM ISO), and used three biological replicates for RNA sequencing (RNA-seq). DEGs were identified according to the criteria of *p* < 0.05 and |log2 FC| > 1 as a measure of significance for DEGs. Volcano plots (Fig. 3a,b) show the DEG expression profiles of the H_2_O_2_-alone vs. control groups and, the ISO group vs. H_2_O_2_-alone group. A total of 2316 DEGs (906 upregulated and 1410 downregulated) in the H_2_O_2_-alone vs. control groups, and 262 DEGs (147 upregulated and 115 downregulated) in the ISO vs. H_2_O_2_ groups were identified.

**Figure 4:**
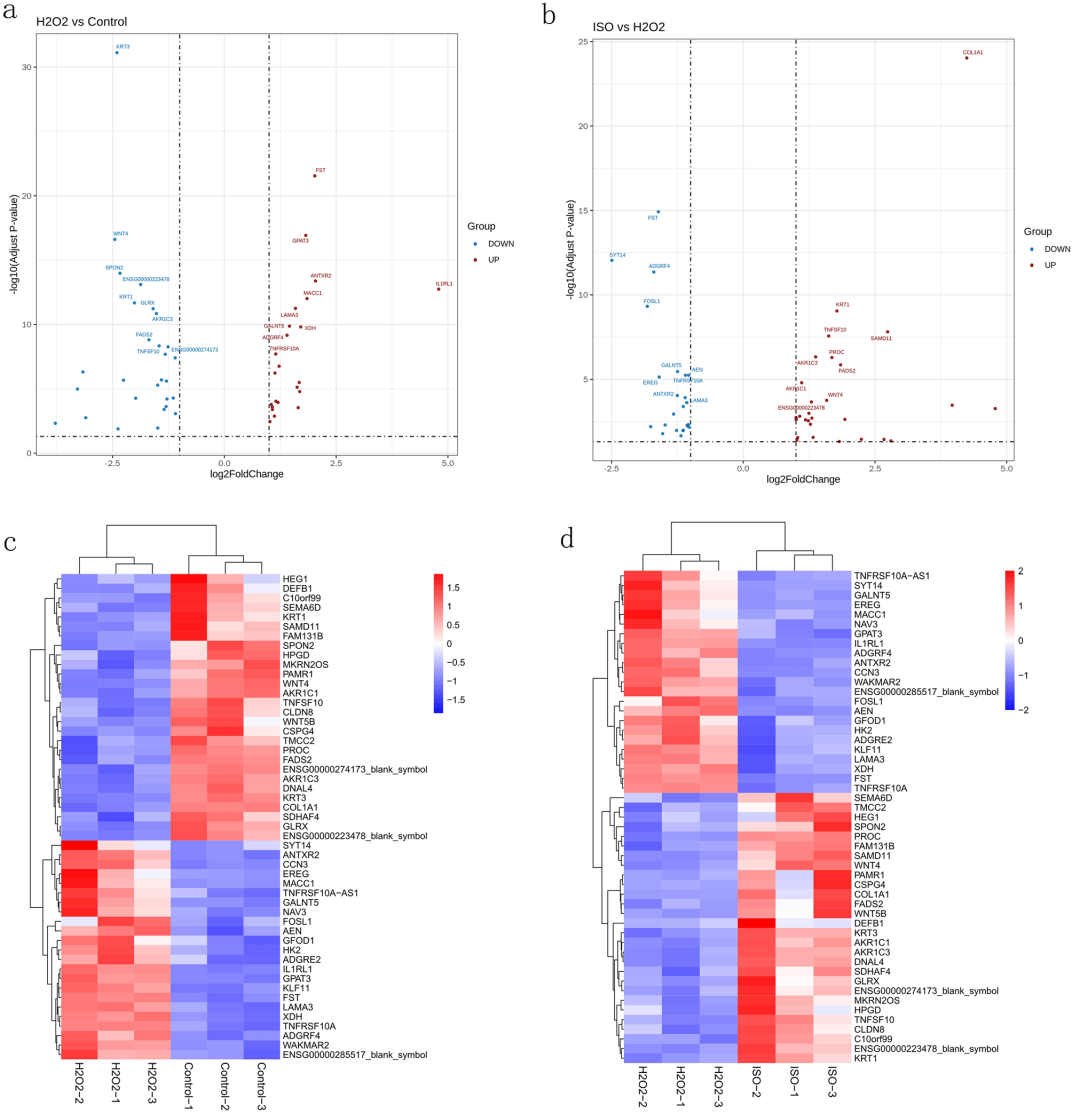
Functional analysis. (**a**,**b**) Volcano plot and (**c**,**d**) Heatmaps of the (**a**,**c**) 23 DEGs that were upregulated by H_2_O_2_ and downregulated by ISO and the (**b**,**d**) 28 DEGs that were downregulated by H_2_O_2_ and upregulated by ISO. The heatmap was generated by TB tools software. *H*_*2*_*O*_*2*_ hydrogen peroxide, *ISO* isorhamnetin, *control group* untreated HaCaT cells, *H*_*2*_*O*_*2*_* group* HaCaT cells treated with 600 μM H_2_O_2_ for 12 h, *ISO group* 600 μM H_2_O_2_ + 40 μM ISO.

A Venn diagram of the identified DEGs is shown in Fig. 3c–e. There were 51 common DEGs between the H_2_O_2_ vs. control and H_2_O_2_ vs. ISO comparison groups (Fig. 3c). Twenty-three genes were upregulated by H_2_O_2_ and downregulated by H_2_O_2_ + ISO (Fig. 3d), including follistatin and glycerol-3-phosphate acyltransferase 3 (Fig. 4a). In addition, 28 genes were downregulated by H_2_O_2_ and upregulated by H_2_O_2_ + ISO (Fig. 3e), including keratin 3 (KRT3) and Wnt family member 4 (WNT4) (Fig. 4b). Treatment with ISO reversed the effect of H_2_O_2_, producing gene expression profiles similar to those observed in the control group. We subsequently performed a functional enrichment analysis using these 51 DEGs. A heat map (Fig. 4c,d) depicts selected DEGs.

**Figure 5:**
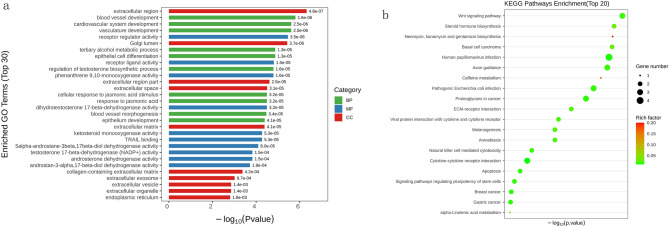
(**a**) Gene Ontology analysis of differentially expressed genes (DEGs) altered by isorhamnetin (ISO). The y-axis shows the GO term, and the x-axis shows the − log10 (*p* value). (**b**) Kyoto Encyclopedia of Genes and Genomes (KEGG) enrichment bubble map of DEGs altered by ISO. The y-axis shows the pathway name, and the x-axis shows the corresponding *p* value of the pathway. The rich factor is the number of DEGs/the total number of genes detected in the pathway.

To gain a better understanding of the potential relationships among these 51 DEGs, we conducted a functional enrichment analysis. Gene Ontology (GO) analysis (Fig. 4a) revealed that the biological processes of the top 30 DEGs, as shown in Fig. 5a, and the most enriched biological processes (BP) were blood vessel development, vasculature development, and tertiary alcohol metabolic process. The most enriched cellular component (CC) terms were receptor regulator activity, receptor ligand activity, and phenanthrene 9,10-monooxygenase activity, and the most enriched molecular function (MF) terms were extracellular region, Golgi lumen, and extracellular region. The top enriched KEGG pathways are shown in Fig. 5b, with the bubble diagram illustrating the top 20 enriched pathways, suggesting that the Wnt signalling pathway; steroid hormone biosynthesis; and neomycin, kanamycin, and gentamicin biosynthesis may be involved in the protective effect of ISO against oxidative stress in HaCaT cells.

### DEG interaction network analysis

We applied the interaction relations in the STRING protein interaction database (http://string-db.org/), and directly extracted the interaction relations of target DEG sets from the database to construct the network in the database. The interaction network diagram of DEGs shows that for the control vs. H_2_O_2_-alone group comparison, the upregulated DEGs protein phosphatase 2 scaffold subunit A beta, signal transducer and activator of transcription 1, and cytoplasmic linker associated protein 2, and the downregulated DEGs mitogen-activated protein kinase 3, adenylate cyclase 6, and histone deacetylase 5 were located in the centre of the network, suggesting that these may be the key genes involved in the oxidative stress-induced damage of HaCaT cells.

## Discussion

*Vernonia anthelmintica* (L.) Willd has been demonstrated to possess several pharmacological properties, including anti-inflammatory, antibacterial, antioxidant, hypoglycaemic, and antithrombotic activities^28^. Its seeds are used in traditional therapy for the treatment of skin diseases, including vitiligo^29^, and its chemical composition is complex; the effective components for vitiligo treatment are mainly flavonoids and caffeic acids^30^. However, current research on vitiligo treatment with *Vernonia anthelmintica* (L.) Willd is mostly limited to the total flavonoid extraction level, and there are few studies on flavonoid monomers. In our previous study, we identified 38 flavonoids from the seeds of *V. anthelmintica* (*L.*) that contain ISO, a bioflavonoid with potential antioxidant properties^15^. To the best of our knowledge, this is the first comprehensive study to reveal the key genes involved in the protective effect of ISO against H_2_O_2_-induced oxidative damage in vitro. Our findings indicated that pre-treatment with ISO markedly increased cell viability in a dose-dependent manner, more than was observed for cells treated with H_2_O_2_ alone. We also found that pre-treatment with ISO reduced the MDA content, enhanced SOD activity, and inhibited apoptosis. Mitochondrial dysfunction is considered an important contributing factor to a variety of pathophysiological situations including aging, heart ischemia/reperfusion injury, diabetes, and cardiovascular diseases, all of which are associated with cell death^31,32^. Mitochondrial dysfunction results in the collapse of the mitochondrial membrane potential, and it has also been shown that the mitochondrial membrane potential decreases in the early stage when stimulated by oxidative stress, and the damage can be reversed by antioxidant treatment^33^. In our study, we found that pre-treatment with ISO reduced the loss of mitochondrial membrane potential, improved cell morphological damage, and inhibited apoptosis in H_2_O_2_-treated HaCaT cells. This indicates that ISO reverses the mitochondrial damage to some extent and sheds new light on the potential of ISO to serve as an antioxidant therapy for vitiligo.

We focused on the DEGs affected in both H_2_O_2_-induced HaCaT cells and those pre-treated with ISO. The DEGs observed in our model system of oxidative stress could be helpful for identifying specific biomarkers of oxidative stress-related diseases. In our study, a total of 2316 H_2_O_2_-responsive genes (906 upregulated and 1410 downregulated), and 262 ISO-responsive genes (147 upregulated and 115 downregulated) were detected, and a large number of DEGs were functionally characterised. By comparing the gene expression of the three groups, we determined that 51 DEGs were potentially related to the protective effect of ISO against oxidative stress in HaCaT cells. Of these, three genes were newly discovered, thus, further follow-up studies are warranted for their functional annotation. Regarding the other genes that have been previously annotated, some are involved in oxidative stress: follistatin protects against oxidative stress and apoptosis both in vitro and in vivo in chronic kidney disease^34^. Moreover, interleukin-1 receptor antagonist (*IL-1RA*) was shown to ameliorate pain hypersensitivity, spinal inflammation, and oxidative stress induced by systemic lipopolysaccharides in neonatal rats^35^. *CTRP3* ameliorates fructose-induced metabolic-associated fatty liver disease by inhibiting xanthine oxidase-associated oxidative stress^36^. Some of the identified genes are also involved in apoptosis. Radiation-induced apoptosis enhancing nuclease (*AEN*) cleaves DNA in concert with other apoptotic nucleases, thereby enhancing apoptosis following ionising irradiation^37^, and knockdown of the MET transcriptional regulator MACC1 sensitises cancer cells to death receptor-mediated apoptosis in solid cancers^38^. Similar results were observed in the present study. Elevated levels of *FST, IL1RL1, XDH, AEN*, and *MACC1* were identified as DEGs in the H_2_O_2_-treated group compared to the control group. Notably, the expression of these genes decreased after pre-treatment with ISO. However, there is limited knowledge regarding the potential functions of these dysregulated DEGs. Future studies will contribute to identifying the precise mechanism of these DEGs in the protective effects of ISO against oxidative injury.

Moreover, 51 DEGs were identified as being possibly related to the antioxidant activities of ISO, and the KEGG enrichment function analysis suggested that the protective effect of ISO may be related to the Wnt signalling pathway and steroid hormone biosynthesis. Wnt signalling plays a crucial role in melanocyte stem cell differentiation^39^. Moreover, it has been shown that Wnt/β-catenin signalling plays a pivotal role in cell proliferation, migration, and differentiation in vitiligo pigmentation systems^40^. Regazzetti et al.^41^ demonstrated that in lesional vitiligo, oxidative stress decreases Wnt expression in melanocytes and keratinocytes. They further demonstrated that pharmacological agents that activate the Wnt pathway successfully induced the differentiation of resident stem cells into pre-melanocytes, suggesting that stimulation of Wnt signalling may serve as an adjunct to current therapies for vitiligo^42^. Mei et al. found that the Wnt5a gene in the canonical Wnt/β-catenin pathway can promote melanocyte differentiation and proliferation^43^. The present study showed that ISO might exert an antioxidant effect by influencing the Wnt pathway, with involvement of Wnt family DEGs (*WNT4* and *WNT5B*). These observations may support further clinical exploration of Wnt agonists to repigment vitiligo lesions. Based on data from public databases, the DEG network was visualised for a comprehensive analysis of key genes and gene pathways involved in the counteraction of H_2_O_2_-induced oxidative damage in HaCaT cells by ISO. This approach could suggest new targeted therapies for vitiligo. Furthermore, GO analysis revealed that the most enriched biological process, molecular function, and cellular component terms of these DEGs were the tertiary alcohol metabolic process, receptor regulator activity, and extracellular region, respectively. DEG interaction network analysis suggested that the key genes were closely involved in the oxidative stress damage in HaCaT cells. Future studies are likely to characterise the mechanisms of action of new transcripts and genes in the protective effects of ISO against oxidative injury.

The present investigation had some limitations. First, validation experiments should be conducted to determine whether manipulating the expression of the top candidate DEGs can affect the various functional readouts of ISO-treated and H_2_O_2_-induced HaCaT cells., Further research is necessary to observe potential changes in the apoptotic rate of H_2_O_2_-induced HaCaT cells by manipulating the expression of top DEG candidates. Additionally, DEG changes in animal models of oxidative damage should also be examined. Second, to address the lack of research on the effect of ISO-involved antioxidant pathways, future studies should aim to more accurately demonstrate the antioxidant stress mechanism of ISO. Third, although the protective effect of ISO against H_2_O_2_-induced oxidative damage in HaCaT cells was investigated in the present study, further experiments and collaborative discussions should be held to identify the effect of ISO on H_2_O_2_-induced melanocytes, which would improve the development of ISO as a source of antioxidants and provide a theoretical basis for the targeted therapy of vitiligo.

## Conclusions

We demonstrated the protective effect of ISO against H_2_O_2_-induced oxidative damage in HaCaT cells, including increased cell viability, reduced MDA content, and enhanced SOD activity compared with cells treated with H_2_O_2_ alone. In addition, we found that pre-treatment with ISO reduced the loss of mitochondrial membrane potential, improved cell morphological damage, and inhibited apoptosis in H_2_O_2_-induced HaCaT cells. In addition, changes in the key genes of untreated HaCaT cells vs. H_2_O_2_-induced HaCaT cells with and without ISO intervention were identified. By combining the functional results with those obtained from the bioinformatics analysis, this study highlighted some aberrantly expressed DEGs and gene pathways that might be related to oxidative stress and the antioxidant effect of ISO. One pathway of note was the Wnt signalling pathway.

## Methods

### Chemicals and reagents

H_2_O_2_ solution, RIPA lysis buffer, 3,5-dimethylphenol (DMSO), and phosphate-buffered saline (PBS) were obtained from Sigma (Shanghai, China). ISO (C_16_H_12_O_7_), SOD, MDA, and proliferation and cytotoxicity assay (CCK-8) kits were obtained from Solarbio (Beijing, China). Dulbecco’s modified Eagle medium (DMEM) and foetal bovine serum (FBS) were purchased from BI Company (Ridgefield CT, USA). Penicillin–streptomycin solution was obtained from Hyclone (Logan, UT, USA). Trypticase was purchased from Gibco (Waltham, MA, USA). Trizol was purchased from Magen; trichloromethane and isopropanol were purchased from Sinopharm; and the RNA Nano 6000 Assay Kit, NEBNext Ultra™ RNA Library Prep Kit for Illumina, and AMPure XP system were from Agilent Technologies (Santa Clara, CA, USA), ABclonal (Woburn, MA, USA), and Beckman Coulter (Brea, CA, USA), respectively. The BCA Protein Quantitation Kit was obtained from Thermo Fisher Scientific (Waltham, MA, USA).

### Cell culture and treatment

Human keratinocytes (HaCaT; Kunming Cell Bank, Chinese Academy of Sciences) were cultured in DMEM supplemented with 10% FBS and a 1% antibiotic mixture containing penicillin and streptomycin. The cells were grown at 37 °C in a humidified atmosphere containing 5% CO_2_. The experiments were performed 24 h after the cells were seeded. HaCaT cells (8 × 10^4^ cells/well) were seeded in 96-well plates for 12 h and then treated with various concentrations (300, 600, 900, or 1200 μM) of H_2_O_2_. Similarly, HaCaT cells were pre-treated with various concentrations (20, 40, 60, or 80 μM) of ISO in the medium prepared above for 12 h to determine the optimal treatment concentration. Subsequently, the pre-treated cells were cultured for an additional 12 h with 600 μM H_2_O_2_ to stimulate oxidative damage. Three replicates per group were used for all experiments. Untreated cells and cells treated with H_2_O_2_ alone were used as controls.

### Cell viability assay

The cellular toxicity of H_2_O_2_ and the protective effect of ISO were measured in HaCaT cells using the CCK-8 assay. After treatment with the indicated concentrations of H_2_O_2_ or ISO, 10 μL of the CCK-8 kit solution was added, and the cells were incubated for 2 h at 37 °C. Subsequently, the absorbance was measured using a microplate reader (Thermo Fisher Scientific).

### Biochemical analysis

Cold PBS was used to collect treated and untreated HaCaT cell samples after centrifugation at 157×*g* for 3 min according to the instructions provided in each enzyme kit. The cells were homogenised with cold PBS, and commercial kits were used to obtain the supernatant for the measurement of MDA and SOD activities.

### Cell apoptosis assay

The protective effect of ISO on the oxidative stress-induced apoptosis of HaCaT cells was determined using the Hoechst assay. Cells pre-treated with 20, 40, and 60 μM ISO were used as the experimental group. After treatment of the cells, 5 μg/mL Hoechst staining solution was added, and the cells were incubated for 10–15 min at 25 °C in the dark. Subsequently, 400 μL of 1 × binding buffer was added, mixed, and placed on ice, and the images of the cells were captured within 1 h under an Olympus fluorescence microscope. This experiment was repeated three times.

### SOD activity

SOD activity in HaCaT cells was measured using the nitroblue tetrazolium method. HaCaT cells at the logarithmic growth stage were digested and washed. The cell suspension was centrifuged at 157×*g* for 5 min. HaCaT cells (1 × 10^6^ cells/well) were seeded in 6-well plates for 12 h and grown at 37 °C in a humidified atmosphere containing 5% CO_2_. When the cells adhered to the wells and the density was approximately 80%, the cells were treated with various concentrations (20, 40, or 60 μM) of ISO for 12 h. Subsequently, the pre-treated cells were cultured for an additional 12 h with 600 μM H_2_O_2_ for 12 h. The cell suspension was then mixed thoroughly with a vortex mixer (ice bath; power 20% or 200 W; ultrasound 3-s, 10-s intervals, 30 repeats) and centrifuged at 8000×*g* at 4 °C for 10 min. The reagents were added to the cell suspension, according to the manufacturer’s instructions. Finally, the mixture was thoroughly mixed and cultured at 37 °C for 30 min, placed in a 1-mL glass cuvette, and the maximal absorbance of the mixture was measured at 560 nm using a spectrophotometer. One unit of SOD was defined as the amount of enzyme that caused 50% inhibition.

### MDA assay

The MDA concentration was used to determine lipid peroxidation. The cell suspension was mixed thoroughly with a vortex mixer (ice bath, power 20% or 200 W; ultrasound 3-s, 10-s intervals, 30 repeats) and centrifuged at 8000×*g* at 4 °C for 10 min. The reagents were added to the cell suspension, according to the manufacturer’s instructions. Finally, the mixture was thoroughly mixed and incubated at 100 °C for 60 min, cooled in an ice bath, and centrifuged at 10,000×*g* for 10 min. Two hundred microliters of supernatant were transferred to a microglass cuvette or 96-well plate, and the maximal absorbance was determined at 450 nm, 532 nm, and 600 nm using a spectrophotometer. The MDA content was calculated according to the protein concentration.

### Mitochondrial ultrastructure observation

HaCaT cells were inoculated into 25-cm^2^ culture flasks and cultured to approximately 80% confluency for fusion. Experimental groups were divided into four groups: blank, control, H_2_O_2_-alone, and ISO + H_2_O_2_. The treatment methods for each group were the same as those described above. Cells were collected into 10-mL centrifuge tubes 24 h after routine digestion and centrifuged at 1000×*g* for 5 min. The supernatant was discarded and 300 µL of high-sugar complete medium was added to the cell precipitate for resuspension. The cell suspension was transferred into a 0.5-mL EP tube, centrifuged at 2000×*g* for 8 min, and the supernatant was carefully removed. The EP tube was tilted at 45°, and 2.5% glutaraldehyde solution was slowly added to the tube wall with a pipette tip until the entire EP tube was filled, followed by fixation at 4 °C for 24 h. The fixed specimens were sent to the Electron Microscopy Center of the Basic Laboratory of Xinjiang Medical University for transmission electron microscopy.

### RNA extraction and monitoring of quality

We selected three groups of HaCaT cells for RNA sequencing: untreated cells, 600 μM H_2_O_2_-induced cells, and H_2_O_2_-induced cells pre-treated with 40 μM ISO (pre-treatment with ISO markedly increased cell viability in a dose-dependent manner versus that observed for cells treated with H_2_O_2_ alone. 40 μM ISO was significantly different to 20 μM but there was no difference with 60 μM. The cells pre-treated with 40 μM ISO were used as the protection group). Total RNA was extracted from the cells using TRIzol reagent according to the manufacturer’s instructions. The absorbance ratio at 260–280 nm of the RNA samples was measured with a Nanodrop ND-2000 system (Thermo Fisher Scientific) and the RNA integrity number was determined using an Agilent Bioanalyzer 4150 system (Agilent Technologies). Only high-quality samples according to assessments of RNA purity, concentration, and integrity were used for library construction.

### Library preparation and sequencing

Paired-end libraries were prepared using ABclonal mRNA-seq Lib Prep Kit (ABclonal) following the manufacturer’s instructions. The mRNA was purified from 1 mg total RNA using oligo (dT) magnetic beads, followed by fragmentation using divalent cations at elevated temperatures in ABclonal First Strand Synthesis Reaction Buffer. Subsequently, first-strand cDNA was synthesised with random hexamer primers and reverse transcriptase using mRNA fragments as templates, followed by second-strand cDNA synthesis using DNA polymerase I, RNase H, and dNTPs. The synthesised double-stranded cDNA fragments were then adapter-ligated to prepare the paired-end library. Adaptor-ligated cDNA was used for polymerase chain reaction (PCR) amplification. PCR products were purified (AMPure XP system) and library quality was assessed using an Agilent Bioanalyzer 4150 system. Sequencing was performed using an Illumina Novaseq 6000/MGISEQ-T7 instrument.

### Data analysis

The data generated from the Illumina/BGI platform were used for the bioinformatic analysis. All analyses were performed using an in-house pipeline from Shanghai Applied Protein Technology.

### Quality control

Raw data (or raw reads) of FASTQ format were first processed using in-house Perl scripts. In this step, the adapter sequence was removed, and low-quality reads [the number of lines with a string quality value less than or equal to 25 accounting for more than 60% of the entire read, or greater than 5% Ns (unknown base)] were filtered out to obtain clean reads that could be used for subsequent analysis.

### Quantification of gene expression level and differential expression analysis

Clean reads were separately aligned to the reference genome in orientation mode using HISAT2 software (http://daehwankimlab.github.io/hisat2/) to obtain mapped reads, which were then spliced with Stringtie software (http://ccb.jhu.edu/software/stringtie/). Gffcompare software (http://ccb.jhu.edu/software/stringtie/gffcompare.shtml) was used for comparing the obtained sequences with the reference genome GTF/GFF file to find the original unannotated transcription region, and to discover new transcripts and new genes. FeatureCounts (http://subread.sourceforge.net/) was used to count the read numbers mapped to each gene. The expression level in FPKM of each gene was calculated based on the length of the gene and read counts mapped to the gene. Differential expression analysis was performed using the DESeq2 package (http://bioconductor.org/packages/release/bioc/html/DESeq2.html); differentially expressed genes (DEGs) with |log2 fold change (FC)| > 1 and adjusted *p* value (Padj) < 0.05 were considered significant DEGs.

### Enrichment analysis

The Gene Ontology (GO) and Kyoto Encyclopedia of Genes and Genomes (KEGG) pathway analyses (http://www.kegg.jpkegg/kegg1.html) can explain the functional enrichment of DEGs and clarify the differences between samples at the gene function level^44^. We used the clusterProfiler R software package for GO function enrichment and KEGG pathway enrichment analysis. When *p* < 0.05, it was considered that the GO or KEGG function was significantly enriched. Transcription factors of the DEGs were extracted directly from the AnimalTFDB (http://bioinfo.life.hust.edu.cn/AnimalTFDB/)/PlantTFDB/)/PlantTFDB (http://planttfdb.cbi.pku.edu.cn/) databases.

### Protein–protein interaction (PPI) analysis

PPI analysis was used to evaluate whether there was an interaction between gene products and other proteins. The analysis was based on protein information corresponding to genes. The known and predicted PPIs were obtained from the STRING database (https://www.string-db.org/). For the gene sequences existing in the database, the networks were constructed by extracting the target gene list from the database. For gene sequences not included in the database, the target gene set sequence was first aligned with the reference sample protein sequence contained in the STRING protein interaction database with BLASTx, and then the protein interaction relationship of the reference gene sequences was used to establish an interaction network. rMATS (http://rnaseq-mats.sourceforge.net/index.html), a variable splicing analysis software suitable for RNA-sequencing data, was used for analysis.

### Statistical analysis

SPSS (version 22.0; IBM Corp., Armonk, NY, USA) was used for statistical analysis. Statistical significance was set at *p* < 0.05. DEseq software was used to process the sequencing data. DEGs were identified according to the criteria of *p* < 0.01 and |log2FC)| > 2.

## Data Availability

The data used to support the findings of this study are available from the corresponding author upon request.
